# Combined transcriptome and metabolome analysis revealed pathways involved in improved salt tolerance of *Gossypium hirsutum* L. seedlings in response to exogenous melatonin application

**DOI:** 10.1186/s12870-022-03930-0

**Published:** 2022-11-30

**Authors:** Wei Ren, Li Chen, Zong ming Xie, Xiaofeng Peng

**Affiliations:** 1 State Key Laboratory of Desert and Oasis Ecology, Xinjiang Institute of Ecology and Geography, Chinese Academy of Sciences, Urumqi, 830011 China; 2 China Fukang Station of Desert Ecology, Chinese Academy of Sciences, Fukang, 831505 Xinjiang China; 3 Xinjiang Production & Construction Group Key Laboratory of Crop Germplasm Enhancement and Gene Resources Utilization, Xinjiang Academy of Agricultural and Reclamation Science, Shihezi, 832000, Xinjiang China; 4 Agricultural Science Research Institute of the third division of Xinjiang production and Construction Corps, Tumushuke, 843800 Xinjiang China

**Keywords:** Abiotic stress, Linoleic acid, Na^+^/K^+^ homeostasis: melatonin, Photosynthesis, Phytohormone signaling, Salinity stress, Upland cotton

## Abstract

**Background:**

Salinization is major abiotic stress limiting cotton production. Melatonin (MT) has been implicated in salt stress tolerance in multiple crops including upland cotton. Here, we explored the transcriptomic and metabolomic response of a salt-tolerant self-bred high-yielding cotton line SDS-01, which was exogenously sprayed with four MT concentrations (50, 100, 200, and 500 μM).

**Results:**

Here we found that MT improves plant biomass and growth under salt stress. The combined transcriptome sequencing and metabolome profiling approach revealed that photosynthetic efficiency is improved by increasing the expressions of chlorophyll metabolism and antenna proteins in MT-treated seedlings. Additionally, linoleic acid and flavonoid biosynthesis were improved after MT treatment. The Na^+^/K^+^ homeostasis-related genes were increasingly expressed in salt-stressed seedlings treated with MT as compared to the ones experiencing only salt stress. Melatonin treatment activated a cascade of plant-hormone signal transduction and reactive oxygen scavenging genes to alleviate the detrimental effects of salt stress. The global metabolome profile revealed an increased accumulation of flavonoids, organic acids, amino acids and derivatives, saccharides, and phenolic acids in MT-treated seedlings. Interestingly, N, N′-Diferuloylputrescine a known antioxidative compound was highly accumulated after MT treatment.

**Conclusion:**

Collectively, our study concludes that MT is a salt stress regulator in upland cotton and alleviates salt-stress effects by modulating the expressions of photosynthesis (and related pathways), flavonoid, ROS scavenging, hormone signaling, linoleic acid metabolism, and ion homeostasis-related genes.

**Supplementary Information:**

The online version contains supplementary material available at 10.1186/s12870-022-03930-0.

## Background

Salinization has become one of the major abiotic stresses restricting agricultural development in arid areas. Climate change, persistent droughts, and rising sea levels are expected to increase salinization [1]. Studies have shown that 424 million hectares of topsoil and 833 million hectares of subsoil of the total global mapped land are salt-affected [2]. Other reports indicated that the global annual cost of salt-induced land degradation in irrigated areas could be US$ 27.3 billion [3]. In China, Xinjiang’s 32.07% of the arable land is affected by salinity [4]. Xinjiang is one of the major areas for upland and sea-island cotton production as 2.5 million hectares were grown in 2022 (http://english.china-cotton.org/, accessed on June 23, 2022). For this province, cotton is a major cash crop; 24% of total cotton production in China is from this region (https://ipad.fas.usda.gov, accessed on June 23, 2022). Even though cotton is classified as a moderately salt-tolerant crop with a salinity threshold level of 7.7 dS/m [5], salinity-driven yield losses together with drought cause hundreds of billions of Yuans of annual losses. More than 9% of the total annual cotton production is lost due to salinity stress [6] which is a serious threat to the sustainable cotton industry in Xinjiang.

Higher soil alkali content significantly affects cotton germination, emergence, and young seedling stages as compared to fully-developed/juvenile plants [7–9]. The poor seed germination in turn affects crop yield. This is because salinity stress in the early stages affects multiple plant organs and tissues such as reduced root growth, lower number of secondary roots, root/shoot ratio, delayed and/or reduced flowering, number of bolls, and boll size [5]. At physiological levels, salt stress decreases the rate of photosynthesis (which is associated with the reduction in chlorophyll and carotenoids) [10]. Salt stress in cotton has also shown changes in the concentrations of inorganic ions such as Mg^2+^, K^+^, and Ca^2+^ [5]. On a molecular level, the salt stress is sensed as osmotic change together with salt-specific calcium waves, halotropism, and inter/extra-cellular sodium sensing. When salt stress is sensed, the cytosolic Ca^2+^ concentration is increased, reactive oxygen species (ROS) are produced, and 3,5-cyclic guanosine monophosphate (cGMP) is increasingly produced ([11] and references therein). The salt overlay sensitive (SOS) pathway is the best-characterized pathway that involves calcineurin B-like proteins (CBLs) and CBL-interacting protein kinases (CIPKs) to coordinate a set of cellular responses to salt stress [12, 13]. Whereas, RESPIRATORY BURST OXIDASE HOMOLOGs (RBOHs) produce ROS waves during salt stress. The Ca^2+^ signals and ROS crosstalk spreads [14] signals between cells. Additionally, phospholipid and protein kinase signaling pathways are also activated during salt stress [15].

There are several ways through which plant responds to salt stress such as accumulation of osmoprotectants, maintaining sodium/potassium homeostasis, increased activity of antioxidants, osmotic adjustments, and production and transport of phytohormones [5, 11]. To manage salt stress, different strategies have been adopted in cotton such as the identification of molecular factors in response to salt stress [16], marker-assisted selection [17], transgenic approaches [18, 19], seed priming, nutrient management [20], and application of chemical compounds that can activate antioxidant systems, improve photosynthesis, promote ion homeostasis, and regulate plant hormone signaling and biosynthesis [21].

Melatonin is a multiple-function molecule that can regulate a range of pathways in plant growth and development. Melatonin has many physiological functions similar to indole-3-acetic acid (IAA) [22]. When applied during salt stress, it significantly reduces salinity-induced ROS by acting as a free radical scavenger as well as an antioxidant [23]. Furthermore, it regulates photosynthesis by alleviating stomatal limitation, increasing chlorophyll contents, and decreasing chlorophyll degradation rates [24]. Its application in plants has to promote ionic homeostasis i.e., higher K+/N+ ratio by upregulating NHX, SOS, and AKT genes [25]. Since it shares similarities with IAA, it can act as a growth regulator. Furthermore, melatonin (MT) has been reported to mediate reduced abscisic acid (ABA) biosynthesis and metabolism by downregulating ABA biosynthesis-related genes. The mediation of the nitric oxide (NO) signaling pathway and polyamine metabolism are effective salt-tolerance mechanisms in salt-stressed plants [26, 27]. Earlier studies on cotton have shown that exogenous MT accelerates seed germination [28], osmotic regulation [29], removes active oxygen, and protects photosynthetic organs [30]. Multi-omics studies in rice [31], olive [32], okra [33], and common bean [34] have shown the participating mechanisms that enable MT-treated plants to tolerate salinity. Some developments have also been seen in cotton genomics where seed priming with MT has been studied [35]. However, the key transcriptomic changes and metabolites that are produced in response to exogenous MT treatment are not known in cotton. Here we treated salt-affected cotton seedlings with 50-500 μM MT solution, identified the most suitable MT concentration, and explored the key transcriptomic and metabolomic changes.

## Material and methods

### Plant material

A self-bred high-yielding upland cotton (*Gossypium hirsutum* L.) line SDS-01 was used as plant material. The plant material has been created by researchers at the College of Agriculture, Xinjiang Agricultural University, China and the seeds used in this study were obtained from them. The selection of SDS-01 line for this experiment is based on our pilot experiments, where it was categorized as a relatively salinity resistant line (data not shown). No permission is required to work on this species. Voucher specimens is available in the genebank herbarium of Xinjiang Agricultural University under the number: XTK1072A87. Official identification of the plant material was conducted by Prof YanPing Ren. The experiment was conducted from late December 2021 to January 2022 at the Nanfan Test Base, Liguo Town, Ledong County, Hainan, China. Plastic pots (25 cm diameter and 27 cm height) filled with 5 Kg soil were used for the experiment. The soil was obtained from local mountains and was rich in organic matter. Twelve seeds were sown in each pot and when the seedlings reached the fourth leaf stage, 10 plants showing similar growth patterns were kept for experimentation. Separate potted plants were sprayed with 100 mL of 50 μM (B1), 100 μM (B2), 200 μM (B3), and 500 μM (B4) MT solution as reported earlier by Jiang, Lu, Liu, Duan, Meng, Li, Zhang, Sun, Zhang and Dong [30]. On the third day of the first MT treatment, 1000 mL of 4% sodium chloride solution was added to achieve a salt content of 0.8%. The negative control (CK) plants were not sprayed with MT and only salt solution was applied. The positive control (CK+) was neither sprayed with MT nor supplied with sodium chloride solution and was only fed with distilled water. The seedling mortality rate, height, growth, and above-ground biomass were measured on the 10th day of stress. The whole procedure was replicated three times. Nine replicates of leaves were collected from each treatment and CK group, washed twice with distilled water, immediately frozen in liquid nitrogen, and stored at − 80 °C. Three replicates of each group were used for transcriptome analysis, qRT-PCR analyses, and metabolome analyses.

### Transcriptome analyses

- RNA extraction, cDNA library preparation, and Illumina sequencing

Total RNAs were extracted from the triplicate samples for CK and B1, B2, B3, and B4 seedlings. The purity, quantity, and integrity of the extracted RNAs was checked on NanoPhotometer spectrophotometer (IMPLEN, Los Angeles, CA, USA), Qubit RNA Assay Kit in Qubit 2.0 Flurometer (Life Technologies, Carlsbad, CA, USA), and RNA Nano 6000 Assay Kit for the Agilent Bioanalyzer 2100 system (Agilent Technologies, Santa Clara, CA, USA), respectively. The RNA extraction, cDNA preparation, and Illumina sequencing were done as reported earlier by [36]. However, the sequencing was performed on an Illumina platform using the PE150 sequencing strategy.

b. Bioinformatics analyses

Raw reads were processed to obtain clean reads. The sequencing error rate was determined, GC (guanine-cytosine) content distribution calculated, low-quality sequences removed, and adaptors decontaminated. The filtered sequences of each sample were aligned with the reference genome [37] to map them to the genome using HISAT2 [38]. New genes were discovered using Cuffcompare [39]. All genes (including new and original annotated genes) were annotated by using BLAST [40] to compare transcripts sequences with KEGG [41], NR [42], Swiss-Prot [43], GO [44], COG/KOG [45], Trembl databases [46].

Fragments Per Kilobase of transcript per Million fragments mapped (FPKM) was used as an indicator of gene expression quantification. These data were then used to determine the overall distribution of sample gene expression. Furthermore, we Pearson’s Correlation Coefficient (PCC) and Principal Component Analysis (PCA) using prcomp and cor functions in R (www.r-project.org (accessed on May 05, 2022). The Venn diagrams and heatmaps were prepared in Interactivenn [47] and TBtools [48], respectively. Differentially expressed transcripts/genes (DETs/DEGs) were identified using DESeq. Fold Change ≥2 and padj < 0.05 were used as screening criteria. Benjamini-Hochberg correction method was used and the resulting significant *p*-values were corrected and finally, padj was used as a key indicator for differentially expressed genes screening. The Plant Transcription Factor Database [49] was used to identify the transcription factors (TFs).

### qRT-PCR analysis

We performed quantitative real-time PCR analysis for 12 genes of high interest (Table 1). The first strand cDNA synthesis from 100 ng of total RNA was achieved as described in section 2.2. The total RNAs were extracted from independent three biological replicates of the treatment and CK groups. Primers were designed using Primer3Plus [50] (Table 1). The 18S RNA (XM_016849259) [51] was used as a reference gene. The reactions were carried out on a Rotor-Gene 6000 machine (Qiagen, Shanghai, China). We used QuantiNova SYBER Green PCR Kit (Qiagen, Shanghai, China. Total reaction volume was 10 μL; 5 μL MonScriptTM RTIII All-in-one Mix with dsDNase (Monad Biotech Co., Ltd.), 0.7 μL of forward and reverse primer each, 2.8 μL RNase-free water, and 1 μL of template DNA. The reaction conditions on the thermal cycler were as follows. 95 °C for 2 min and 40 cycles of 95 °C for 10 mi and 60 °C for 30 sec. Three biological replicates were analyzed in independent runs. Relative gene expressions were calculated by using 2^^-ΔΔct^ method [52]. The graphs for the genes’ expressions were prepared in Microsoft Excel 2019®.

**Table 1:** List of primers used for qRT-PCR analysis

Gene name		sequence(5′-3′)
*gene-LOC107940220; chlorophyllase-1*	F	TCGTCACACCTTCCGATGAA
	R	TAGGAGGCAATATATACAGCTGAGG
*gene-LOC107932194; photosystem I chlorophyll a/b-binding protein 6*	F	GATGTGGGGAAGAGGGTCAC
	R	TGTGTTGTGAAAGCCGAGAAA
*Gene-LOC121229296; superoxide dismutase*	F	CGTGGACAAGCAGATACCACT
	R	TGCCAATCTTCCACCTGCAT
*gene-LOC107898050; glutathione S-transferase F8*	F	AAGGGCAGAGCTTTAACCCC
	R	ACACCTCCAGCACTTTAGCC
*gene-LOC107908901; ABA-INSENSITIVE 5-like protein 7*	F	ACCAGTTCCTTACATATTCGGC
	R	GAGTTCCAATGTATAGGCCTGC
*gene-LOC107958111; BRI1 kinase inhibitor 1-like*	F	TCGGGAATCGTCCTCAAAGC
	R	CCGAACCATCCTCACGTACC
*gene-LOC121208278; cyclin-D3–3-like isoform X2*	F	CTCAAGACATGTGAGGATGAAGT
	R	ACCGAAGATATCGCAGTCCA
*Gene-LOC107900395; cytochrome c6, chloroplastic*	F	TCTCCTATCTGCAACACCCCA
	R	CAGTGACAACCCCATTCCTTTC
*gene-LOC107926059; Oxygen-evolving enhancer protein*	F	AGACATCCATGAGTTGGGGC
	R	CCCATCACTTGTTCTCTCTTGC
*gene-LOC107935797; cation/H(+) antiporter 15-like*	F	CAGTTGACCCTGGTGGTTGT
	R	TGGACCTAATAATACTCCTCCGAGA
*gene-LOC107923245; cation/H(+) antiporter 2-like*	F	CCACCCTTGCCGCTTGTTA
	R	TTTTCCCACAGCATTTTGGGT
*gene-LOC107888574; phosphate transporter PHO1 homolog 9-like*	F	TCCCTTGTTCCTGCTCATTGTT
	R	GAAATCTGGGAGAGTGACCTTGT
*18S rRNA*	F	TTACGCAATGCGCTCTGGA
	R	ACCGCAGAGCTGACAGATG

### Sample preparation and metabolome analyses

The washed seedlings stored at −80 °C were used for metabolome analyses. The triplicate samples for each treatment and CK were freeze-dried by a Scientz-100F vacuum freeze-dryer and crushed for 1.5 min at 30 Hz in an MM400 mixer mill using zirconia beads. The lyophilized sample powder (100 mg) was then dissolved in 1.2 ml methanol (70%) by vertexing for 30 seconds. This step was repeated six times followed by overnight refrigeration of the samples at 4 °C. Next day, the samples were centrifuged at 12000 rpm for 10 min and the extracts were filtrated (SCAA-104, 0.22 μm pore size; ANPEL, Shanghai, China) before UPLC-MS/MS analysis.

The extracted triplicate samples were analyzed in an LC-ESI-MS/MS system (UPLC, Shim-pack UFLC SHIMADZU CBM A system; MS, QTRAP® 4500+ System). For analysis, the UPLC column was Waters ACQUITY UPLC HSS T3 C18 (1.8 μm, 2.1 mm*100 mm), column temperature of 40 °C, and flow rate of 0.4 mL/min. The injection volume for samples was 2 μL. The sample system consisted of water (0.1% formic acid) and acetonitrile (0.1% formic acid). The gradient program was set as 95:5 V/V at 0 min, 5:95 V/V at 10.0 min, 5:95 V /V at 11.0 min, 95:5 V/V at 11.1 min, and 95:5 V/V at 15.0 min. For ESI-Q TRAP-MS/MS we used the instrument, settings, conditions, and software as reported by Li*,* et al. [53].

The metabolites were analyzed based on the NMDB database (Norminkoda Biotechnology Co., Ltd. Wuhan, China) and other public databases as reported previously Li, Chen, Duan, Zhao, Zhang, Zang and Ya [53]. Mass spectral data were processed in Analyst 1.6.3 (Sciex, Framingham, MA, USA). The metabolite quantification was done in the MRM mode of QQQ MS. Once the metabolite MS data was obtained, we used MultiQuant (3.0.2, AB SCIEX, Concord, ON, Canada) for peak area integration, followed by the determination of the relative metabolite contents using chromatographic peak area.

The unsupervised PCA and PCC were computed using prcomp and cor functions in R (www.r-project.org (accessed on May 05, 2022). Orthogonal partial least squares discriminant analysis (OLPS-DA) was performed for the identified metabolites and the differentially accumulated metabolites (DAMs) were identified between the CK and treatment groups. The screening conditions for DAMs identification between the groups were as follows. Fold change ≥1.5 and ≤ 0.67 and variable importance of projection (VIP) ≥1. The VIP values were extracted from OPLS-DA results done in the R package MetaboAnalystR (https://github.com/xia-lab/MetaboAnalystR (accessed on May 5, 2022)). The data were log-transformed (log2) and mean-centered before OPLS-DA. To avoid overfitting, a permutation test (200 permutations) was performed.

Metabolite annotation was done in the KEGG compound database (http://www.kegg.jp/kegg/compound/ (accessed on May 10, 2022)). The metabolites which could be annotated were then mapped to the KEGG Pathway database (http://www.kegg.jp/kegg/pathway.html (accessed on May 10, 2022)). The pathways to which the DAMs could be significantly mapped were entered in metabolite sets enrichment analysis (MSEA), followed by the determination of their significance using the hypergeometric test’s *p*-values.

## Results

### Exogenous melatonin application improves salt stress tolerance in upland cotton seedlings

**Fig. 1:**
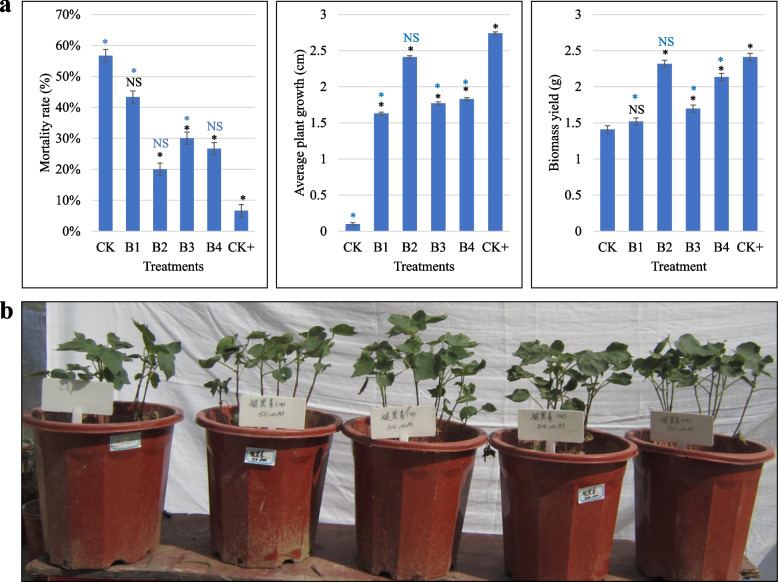
**a** Morphological characteristics i.e., the mortality rate (%), average plant growth (cm), and biomass yield (%), of cotton seedlings exposed to salt stress and treated with melatonin. The error bars show the standard deviation. The black marks show the least significant differences between the treatments and CK. The blue marks show the least significant differences between CK+ and the treatments. * = significant and NS = non-significant differences when *p-*value is 0.05. **b** Phenotypes of the seedlings exposed to salt stress and treated with different melatonin concentrations. Where CK = 0.8% salt stressed, B1, B2, B3, and B4 = 0.8% salt-stressed seedlings exogenously sprayed with 100 ml of 50 μM, 100 μM, 200 μM, and 500 μM melatonin solution, respectively, and CK+ = no salt treatment and no melatonin treatment

Cotton seedlings responded differently under CK (control), B1 (50 μM), B2 (100 μM), B3 (200 μM), and B4 (500 μM). Compared to CK, B2, B3, and B4 differed significantly for mortality rate i.e., CK had the highest mortality rate, whereas the mortality rate decreased (generally) with an increase in MT concentration. However, B3 and B4 showed relatively higher mortality as compared to B2, indicating that higher concentrations may not be necessary for relieving salt-stress. The CK seedlings showed stunted growth as evident from the least average plant growth (cm), whereas the MT treated showed significant average plant growth such that increasing MT helped the seedling to increase average plant growth. Finally, we observed that CK showed the least biomass yield (g) as compared to MT-treated ones. The biomass yields also showed a continuous increase with the increasing MT. Interestingly, we noticed that the B2 treatment didn’t follow the concentration trend in all three characteristics such that the mortality rate for B2 was the lowest of all MT treatments. Similarly, B2 showed higher average plant growth and biomass yield than B1 and B3 but lower than B4 (Fig. 1). These observations indicate that B2 is ideal concentration to treat cotton seedlings to overcome the applied salt stress.

### Transcriptome profile of melatonin-treated cotton seedlings exposed to salt stress

**Fig. 2:**
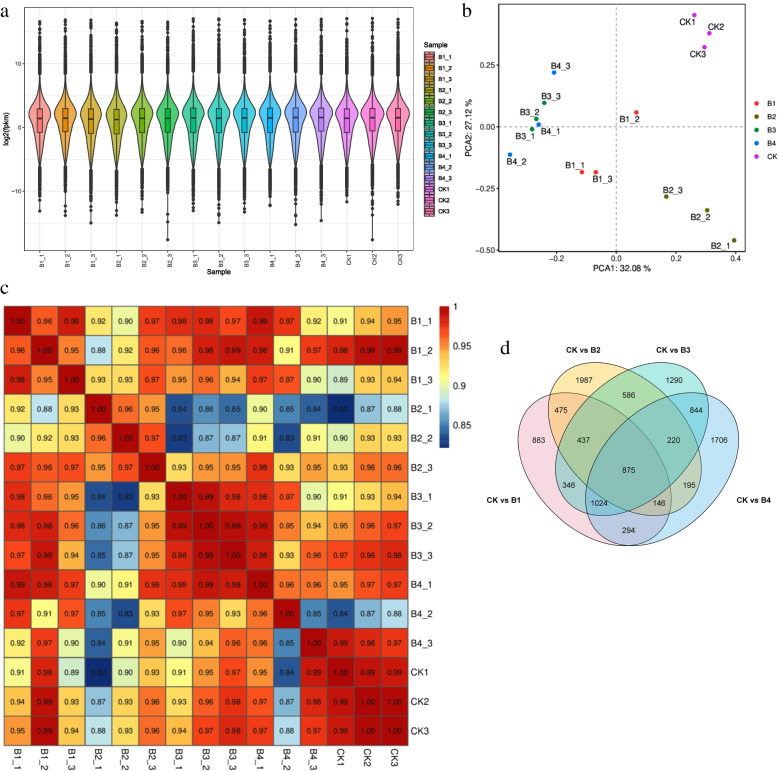
**a** Violin boxplot representing overall fpkm values for each replicate. The abscissa represents different samples, the ordinate represents the logarithm (log2) of the FPKM of the sample expression. The graph measures the expression level of each sample from the perspective of the overall dispersion of expression levels. **b** Principal component analysis of the sample gene expression. Different colors represent different samples. **c** Pearson correlation coefficient of the sample gene expression represented as a heatmap. **d** Venn diagram representing the number of differentially expressed genes between different treatments vs CK. Where CK = 0.8% salt stressed, B1, B2, B3, and B4 = 0.8% salt-stressed seedlings exogenously sprayed with 100 ml of 50 μM, 100 μM, 200 μM, and 500 μM melatonin solution, respectively. 1, 2, and 3 with the treatments represent the replicates

The global transcriptome changes in the cotton seedlings in response to exogenous application of different MT concentrations were studied by RNA sequencing. A total of 15 libraries were sequenced which resulted in an average of 52,316,506 reads per library. The clean read ranged from 93.6 to 98.87% with an average GC content of 45%, Q20 of 97%, and Q30 of 92% (Additional file 1: Supplementary Table 1). A total of 305 new genes were identified from the 15 libraries’ data, making a total of 67,690 genes that could be annotated in Swiss-Prot, GO, eggNOG/COG, KOG, KEGG, and Pfam. CK showed higher overall gene expression than B1-B4 (Fig. 2 a). The PCA analysis showed that treatment replicates tended to group (Fig. 2 b). Similarly, the PCC ranged from 0.85 to 1 (Fig. 2 c). Thus, the PCA and PCC indicate that the sampling was reliable.

- Differential gene expression between melatonin treated and CK cotton seedlings exposed to salt stress

A total of 5485, 5501, 6774, and 6299 transcripts were differentially expressed in treatment comparisons CK vs B1, CK vs B2, CK vs B3, and CK vs B4, respectively (Fig. 2 d). The DEGs in CKvsB1 and CKvsB3 were enriched in protein processing and endoplasmic reticulum, MAPK signaling pathway, alpha-linolenic acid metabolism, linoleic acid metabolism, flavonoid biosynthesis, phenylpropanoid biosynthesis, and photosynthesis-related pathways.

b. Melatonin upregulates photosynthesis and chlorophyll biosynthesis genes in salt-stressed cotton seedlings

**Fig. 3:**
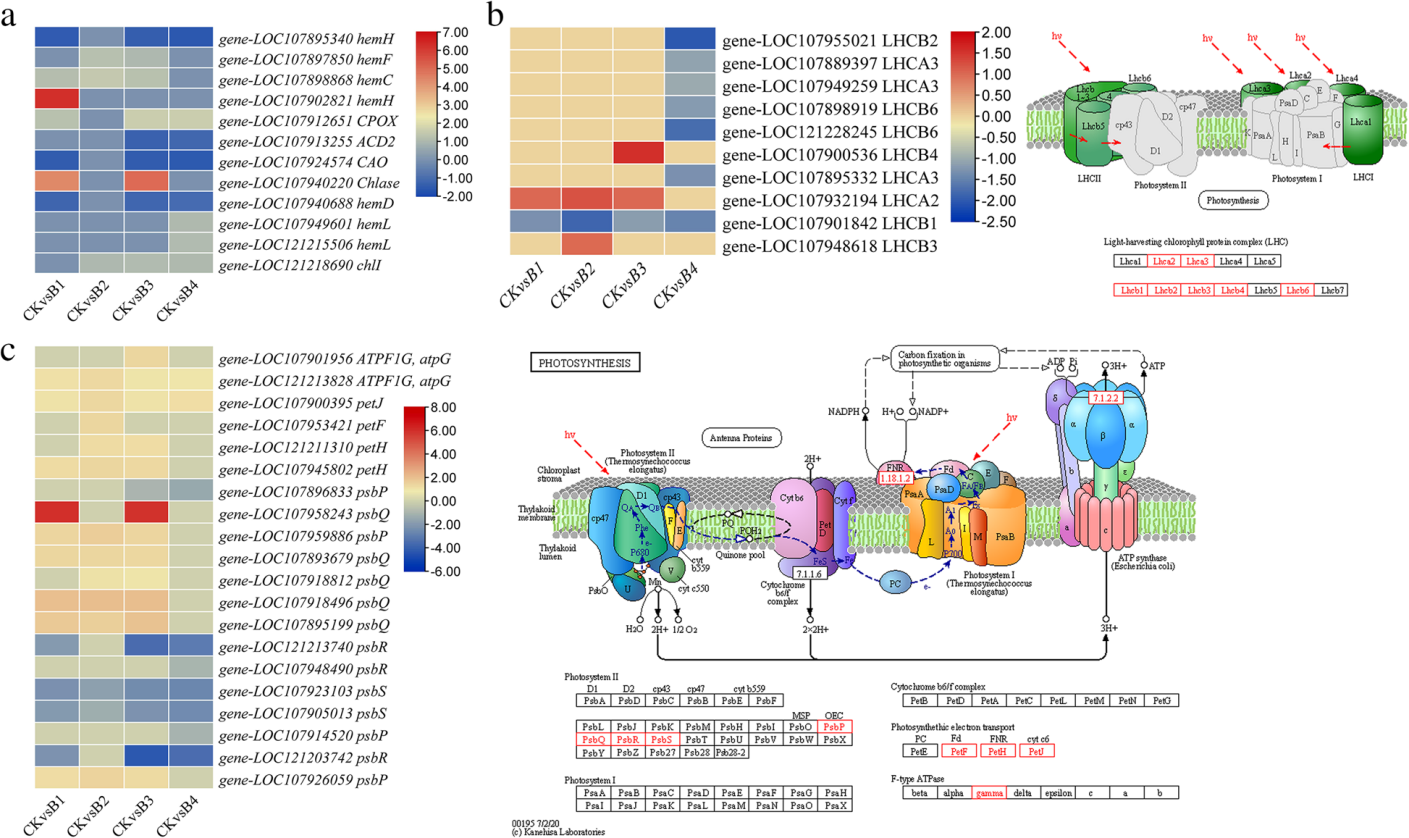
**a** Heatmap of log2FC values of the DEGs enriched in porphyrin and chlorophyll metabolism pathway (Ko00860). **b** Heatmap and pathway map of the DEGs enriched in photosynthesis – antenna proteins (Ko00196). **c** Heatmap and pathway map of the DEGs enriched in photosynthesis (Ko00195). The second panels in “b” and “c” show the respective KEGG pathway maps with the red boxes representing the enriched DEGs. The maps were developed in the KEGG pathway database [54] using the KEGG pathway mapper [55] by searching Ko IDs of the DEGs on respective pathways. Where CK = 0.8% salt stressed, B1, B2, B3, and B4 = 0.8% salt-stressed seedlings exogenously sprayed with 100 ml of 50 μM, 100 μM, 200 μM, and 500 μM melatonin solution, respectively

Twelve DEGs were enriched in the porphyrin and chlorophyll metabolism pathway (Fig. 3 a; Additional file 1: Supplementary Table 2). A chlorophyllase-1 (*gene-LOC107940220*) transcript was upregulated in B1 and B3 indicating higher chlorophyll biosynthesis as compared to CK. Additionally, coproporphyrinogen-III oxidase 1 (*hemF*) showed upregulation in response to MT treatment as compared to CK. A ferrochelatase-2 (*hemH, gene-LOC107902821*) was exclusively expressed in B1 as compared to CK. Two *hemL* transcripts (glutamate-1-semialdehyde 2,1-aminomutase 2) showed exclusive expression in B4 and were upregulated as compared to CK. Furthermore, a magnesium-chelatase (*ChlI, gene-LOC121218690*) was upregulated in MT-treated seedlings as compared to CK (except B1). Finally, the expression of a porphobilinogen deaminase (*hemC*, *gene-LOC107898868*) increased in B1-B3 as compared to CK, whereas it wasn’t expressed in B4. Overall, these changes indicate higher chlorophyll biosynthesis in MT-treated cotton seedlings as compared to CK.

Since chlorophyll biosynthesis genes showed increased expressions in MT-treated seedlings, therefore, we further checked for photosynthesis and antenna proteins related genes. Ten DEGs were enriched in the photosynthesis – antenna proteins pathway (Fig. 3 b; Additional file 1: Supplementary Table 2). These DEGs showed downregulation in B4 as compared to CK. A chlorophyll a-b binding protein CP29.3 (*LHCB4, gene-LOC107900536*) was upregulated in B3 as compared to CK but not expressed in other treatments. Interestingly, a photosystem I chlorophyll a/b-binding protein 6 (*LHCA2, gene-LOC107932194*) showed upregulation in all treatments except B4, in which it was not expressed. Finally, an *LHCB3* (*gene-LOC107948618*) was upregulated in B2 as compared to CK and not expressed in others. These changes suggest that LHCB4, LHCB3, and LHCA2 are upregulated in response to MT treatment.

Finally, there were 20 DEGs enriched in photosynthesis (Fig. 3 c; Additional file 1: Supplementary Table 2). Two F-type H^+^-transporting ATPase (ATPF0A), a cytochrome c6 (petJ), a ferredoxin (petF), two ferredoxin--NADP+ reductases (petH), four photosystem II oxygen-evolving enhancer protein 2 (PsbP), five photosystem II oxygen-evolving enhancer protein 3 (PsbQ), and a photosystem II 10 kDa protein (PsbR) were upregulated in MT treated seedlings as compared to CK. The petF was B2 specific, while petHs and PsbQs were not expressed at all in B4. Two PsbRs and two photosystem II 22 kDa proteins (PsbS) showed downregulations in all treatments; the PsbRs didn’t express at all in B2. Overall, we observed that MT triggered the upregulation of most of the photosynthesis proteins involved in proton pumping, electron transport chain, and photosystem II. These observations indicate that MT improved photosynthesis.

iii. Melatonin significantly alters the phytohormone signaling pathway in salt-stressed cotton seedlings

Two hundred and 20 DEGs enriched in the plant-hormone signal transduction pathway could be annotated as 33 genes. Of these, 98 were related to auxin signaling. The auxin influx carrier (AUX1) transcripts showed changes in expression in response to B2 and B3 treatments. Nine (B1), 15 (B2), 27 (B3), and 14 (B4) AUX/IAA proteins were downregulated as compared to CK suggesting their degradation. Similarly, ARFs were also downregulated in MT-treated seedlings as compared to CK. Whereas, the genes that are affected by ARF activity i.e., IAA-amido synthetase (GH3) and auxin-responsive proteins SUAR (SUAR) showed mixed expression as compared to CK (Additional file 1: Supplementary Table 2).

Twelve transcripts annotated as four cytokinin signaling-related genes were differentially expressed between the studied treatments. The cytokinin receptor histidine kinases (HKs) were not expressed in B1 and B2, while upregulated in B3 and B4 as compared to CK. Similarly, two-component response regulators (ARR2-like and ORR24-like) were upregulated in MT-treated seedlings except for B2 where they didn’t show any expression. On the contrary, the ARR17, ARR8-like, and ARR9-like transcripts showed downregulation in MT-treated seedlings as compared to CK. These changes show that cytokinin is perceived and its signal output is observed when salt-stressed cotton seedlings are sprayed with MT. For gibberellin signaling, eight transcripts were differentially expressed. Gibberellin receptor GID1A was downregulated in B3, a GID1B-like was upregulated in B1 and B3, while another was downregulated in B3, and GIDC-like were downregulated in B3 and B4. Two DELLA proteins (SLR1-like) were upregulated in B1-B3 but not expressed in B4. The downregulation of GID-like and upregulation of SLR1-like genes/proteins indicate that under the influence of salt and the application of different MT concentrations, the GA signals were not strong enough to activate GIDs and degrade DELLAs. This was confirmed by the absence of differential expression of key active GAs biosynthetic genes (Additional file 1: Supplementary Table 2).

The ABA receptor PYL4-like was upregulated in B3, while not expressed in other treatments. The PYL8-like transcripts showed variable expression i.e., downregulated in B1, B3, and B4, while one transcript was upregulated in B2 as compared to CK. Twenty transcripts annotated as protein phosphatase 2C (PP2Cs) were differentially expressed between CK and MT-treated cotton seedlings; all were downregulated. A relatively higher number of transcripts were downregulated in B3 and B4 as compared to B1 and B2. Similarly, the serine/threonine-protein kinase SAPK2-like (SnRK-like) was downregulated in MT-treated seedlings. However, only one transcript was expressed in B1 (downregulated) and none was expressed in B2. The ABSCISIC ACID-INSENSITIVE 5-like protein 7 (ABF7) was only expressed in B1 and B2, while both ABF5 and ABF7 transcripts were downregulated in B3 and B4 (Additional file 1: Supplementary Table 2).

The ethylene receptors (ETRs), serine/threonine-protein kinase CTR1-like (CTR1-like), and EIN3-binding F-box protein 1-like (EBF1), showed reduced expression in B1-B3 but were not expressed in B4. The ethylene response factors (ERFs) were also downregulated in all treatments as compared to CK except in B3 where the only expressed transcript showed downregulation. The downregulation of CTR1-like suggests that its activity is repressed [56]. However, no expression in B4 indicates that higher MT concentration somehow affected the ethylene signaling pathway. As far as brassinosteroid signaling-related genes are concerned, all expressed transcripts annotated in this pathway were upregulated in MT-treated seedlings except the cyclin-D3–1-like (CYCD3–1) transcripts, which were downregulated in B1-B3 and not expressed in B4. Interestingly, brassinosteroid insensitive 1 (*BRI1, gene-LOC107958111*) was upregulated both in B2 and B4. One BKI1 was upregulated in all treatments, while the other two BRI1s (*gene-LOC107940302* and *gene-LOC107905559*) and two BAK1s were B2 specific. These observations indicate that MT affects brassinosteroid signaling with a pronounced effect in B2-treated seedlings. The upregulation of cyclin-D3–3-like isoform (*CYCD3–3, gene-LOC107914329*) in B3 and *gene-LOC121208278* in B1 and B3 is interesting. The downregulation of CYCD1-like transcripts in B2 and B3 indicates that MT treatment arrested the G1 phase. This could also be due to sucrose starvation. While the activation of CYCD3–3 s indicates cell proliferation and secondary growth in said treatments [57] (Additional file 1: Supplementary Table 2).

The DEGs enriched in Jasmonic acid (JA) signaling were downregulated in B2 and B4 but were not expressed in the other two treatments as compared to CK. Especially, the downregulation of MYC2-like TF solely in B2 indicates activation of JA signaling [58]. Similarly, the downregulation of TIFY proteins (TIF 10A in B4, TIFY 9 in B2 and B4) indicates relieving the seedlings from the repression of JA responses [59]. Finally, the transcripts enriched in salicylic acid signaling were also downregulated as compared to CK; TGA1 and TGA2 were expressed in B1, while only TGA2 was expressed in B2. B4 showed expression (though lower than CK) for most of the transcripts. The downregulation of NPR-like proteins indicates relieving of MT-treated seedlings from negative regulation of the transcriptional defense responses [60] (Additional file 1: Supplementary Table 2).

iv. Transcriptomic changes in linoleic acid and α-linoleic acid pathways

The linoleic acid and α-linoleic acid pathways were common in all the treatment comparisons suggesting that these pathways are important in alleviating salt stress effects after exogenous MT application. Forty DETs annotated as 12 genes were enriched in the α-linoleic acid pathway. On the other hand, 12 DETs annotated as four genes were enriched in the linoleic acid pathway. A phospholipase A2 (*PLA2G, gene-LOC107907519*) was exclusively expressed (upregulated) in B4 as compared to CK. Most lipoxygenase transcripts (seven) were upregulated in B1, followed by B2 (three) and B3 (2). Two lipoxygenases (linoleate 13S-lipoxygenase 3–1, *gene-LOC121230896* and *gene-LOC107915240*) were common to B1, B2, and B3 treatments. None was expressed in B4. The allene oxide synthase-like (AOS), allene oxide cyclase (AOC), 12-oxo transcripts, 12-oxophytodienoate reductase 2-like (OPR), 4-coumarate-CoA ligase-like 5 (OPCL5), and peroxisomal acyl-coenzyme A oxidase 1-like (ACX) were upregulated in at least one treatment as compared to CK. Whereases, the 3-hydroxy acyl-CoA dehydrogenase (MFP2) and Jasmonate O-methyltransferase (JAOMT) were downregulated. However, it must be noted that none were expressed in B4. These changes indicate that MT triggers the expression of multiple genes controlling various steps in the biosynthesis of α-linoleic acid. However, the expressions also indicate that JA biosynthesis is limited in B4, which is also evident from the expression of JAZ3 and TIFYs enriched in the plant-hormone signaling pathway.

e. ROS Scavenging

**Fig. 4:**
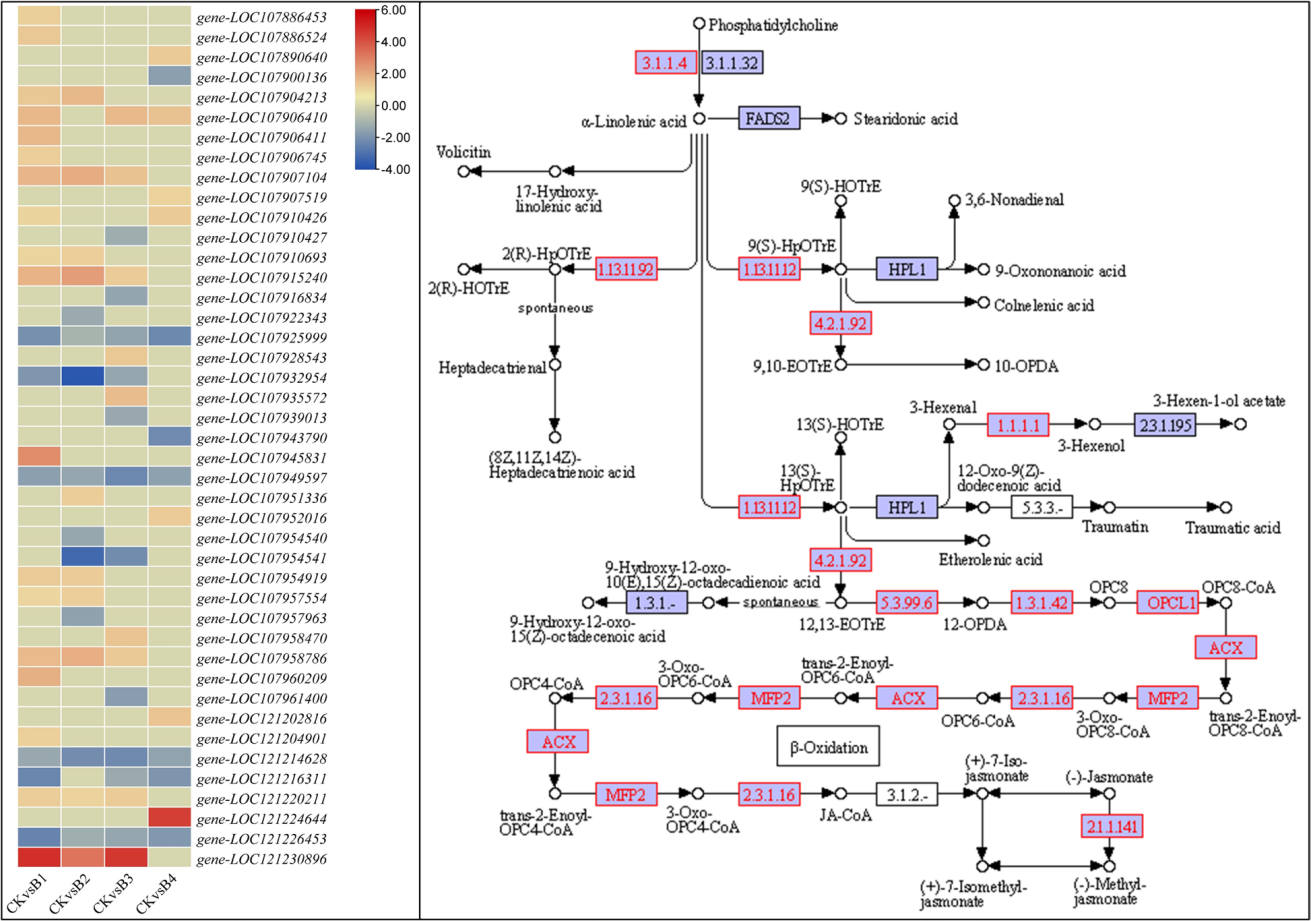
Heatmaps of the log2FC values of the DEGs related to glutathione metabolism pathway (ko00480), reactive oxygen species scavenging, Na^+^ and K^+^ transport. Where CK = 0.8% salt stressed, B1, B2, B3, and B4 = 0.8% salt-stressed seedlings exogenously sprayed with 100 ml of 50 μM, 100 μM, 200 μM, and 500 μM melatonin solution, respectively

As a part of ROS scavenging, we explored the glutathione metabolism as well as other key genes. Eighteen DETs annotated as six genes were enriched in the glutathione metabolism pathway. A leucine aminopeptidase 3 (*pepA, gene-LOC107920609*) was solely upregulated in B3 as compared to CK. A glutamyltranspeptidase 1-like (GGT1) was upregulated in B4, while another was downregulated in B1 as compared to CK. Out of five glucose-6-phosphate 1-dehydrogenase (G6PDs), one (*gene-LOC107886427*) was upregulated in B1, B2, and B3, while two (*gene-LOC107909547* and *gene-LOC107933096*) were specifically upregulated only in B4 (Fig. 4; Additional file 1: Supplementary Table 2). The expression of pepA, GGT1, and G6PD genes indicate that MT application induces relatively higher glutathione and L-glutamate biosynthesis as compared to CK.

Other transcripts like 20 kDa chaperonin were downregulated in B1, B3, and B4 but not expressed in B2. The alpha carbonic anhydrase 1 (ACA1) transcripts were upregulated in B1, while ACA7 was upregulated in B1, B3, and B4. A most interesting result was the upregulation of two superoxide dismutase (SOD) transcripts in B1, B2, and B3, while a third (*gene-LOC121229296*) was upregulated in all treatments. The glutathione s-transferase (GST) F8, 23-like, U17-like, and seven other transcripts showed upregulation in at least one treatment as compared to CK. Only one catalase isozyme 2-like (CAT-2, *gene-LOC107937827*) was upregulated in B1 and B3 and not expressed in B2 and B4. Other GSTs, glutathione peroxidase (GPXs), and ascorbate peroxidase (APXs) were downregulated in one or more treatments. The upregulation of ACA1, ACA7, SODs, and GSTs (F8, 23-like, U17-like) shows that MT treatment enhances the ROS scavenging potential of the salt-stressed cotton seedlings (Fig. 4; Additional file 1: Supplementary Table 2).

f. Transcriptional changes in potassium and sodium transporters

The cation/calcium exchanger 2-like (CCX2) transcripts were upregulated in B4 but downregulated in B1 and B2. Interestingly two CCX5 transcripts (*gene-LOC107937422 and gene-LOC107887692*) showed upregulation in all treatments as compared to CK. The cation/H^+^ antiporters (CHXs) i.e., CHX15 and CHX2 showed increased expression in MT-treated cotton seedlings, while the CHX20 was exclusively upregulated in B3. It is important to note that CHX2 wasn’t expressed in CK and was only expressed after MT treatment. K^+^ efflux antiporter (KEA) KEA3 and KEA5 showed decreased expressions in B1 and B1, B3, and B4, respectively, whereas the KEA4 (*gene-LOC107940206*) was exclusively upregulated in B2 as compared to CK. Two phosphate transporter PHO1 (homolog 3 and 9) showed increased expression in MT treatments. Two of 3 K^+^-channels AKT1 were upregulated in B2 and B3, while a third was upregulated in B1-B3. Potassium transporter 8 transcripts were upregulated in B1-B3. Interestingly, two protein SUPPRESSOR OF K^+^ TRANSPORT GROWTH DEFECT 1 (SKD1) transcripts and two CIPK 9-like transcripts showed exclusive upregulation in B2 as compared to CK (Fig. 4; Additional file 1: Supplementary Table 2).

Eleven other transcripts were found (in addition to CCSs and CHXs) differentially expressed when we searched for keywords sodium in the annotation files. Three of the six vacuolar cation/proton exchanger 3 (CAX3) transcripts were downregulated in at least one treatment, while the other three were upregulated; the first was B4 specific, the second was expressed in B1, B2, and B3, while the third was expressed in B3 and B4. A Na^+^/H^+^ exchanger 7 (NHX7) showed increased expression in MT-treated seedlings except for B2 (Fig. 4; Additional file 1: Supplementary Table 2).

### qRT-PCR analyses

To validate the RNA sequencing results, we studied the expression patterns of 12 important transcripts/genes. The data showed that the genes’ expressions showed similar trend as of transcriptome analyses (Additional file 2: Fig. S1a). This was further confirmed by the PCC > 0.83 (Additional file 2: Fig. S1b). These findings also confirm that their roles in respective pathways is important as presented in above sections.

### Metabolome profile of melatonin-treated cotton seedlings

**Fig. 5:**
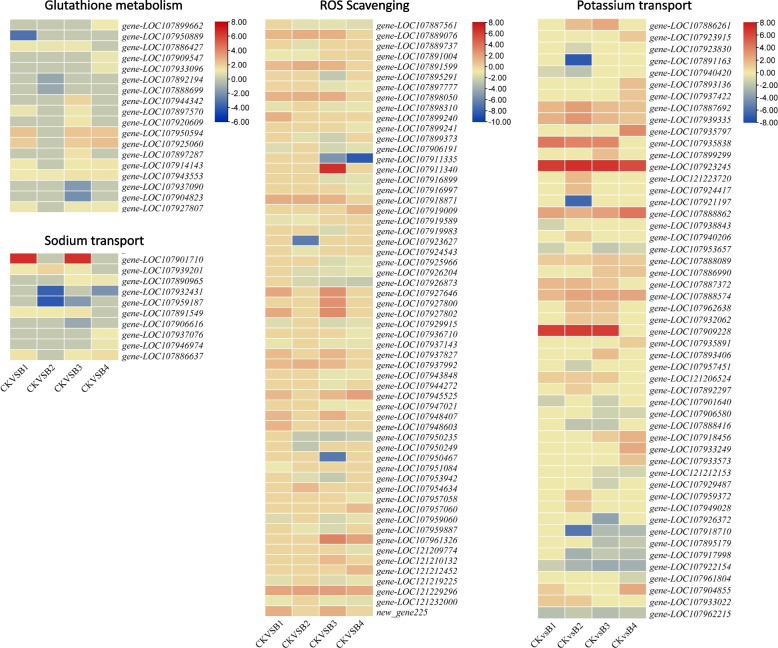
**a** Heatmap of the relative intensities, **b** Venn diagram of the differentially accumulated metabolites between different treatment comparisons, **c** Principal component analyses, and **d** Pearson’s Correlation Coefficient heatmap of the detected metabolites in melatonin treated and control cotton seedlings exposed to salt stress. Where CK = 0.8% salt stressed, B1, B2, B3, and B4 = 0.8% salt-stressed seedlings exogenously sprayed with 100 ml of 50 μM, 100 μM, 200 μM, and 500 μM melatonin solution, respectively. 1, 2, and 3 with the treatments represent the replicates

The UPLC-MS/MS analyses identified 735 metabolites in the studied cotton seedlings (CK and treated with MT) (Fig. 5 a). These metabolites were annotated into 22 compound classes. The comparison of the treated samples with CK revealed the differential accumulation of 227, 225, 167, and 174 metabolites in CKvsB1, CKvsB2, CKvsB3, and CKvsB4, respectively; 85 DAMs were common in all treatment comparisons (Fig. 5 b; Additional file 1: Supplementary Table 3). The PCA analysis showed that the treatment replicates were grouped and the contribution of the first two principal components was 55.17% (Fig. 5 c). The PCC ranged from 0.95 to 1.0 confirming the reproducibility among the biological replicates of all the treatments and CK (Fig. 5 d).

The DAMs were enriched in glutathione metabolism, linoleic acid metabolism, plant-hormone signal transduction pathway, flavonoid biosynthesis, and phenylpropanoid biosynthesis pathways (Additional file 3: Supplementary Fig. 2). Among the common DAMs, are amino acids (glutamic acid, arginine, glutathione (reduced form), and lysine), flavones, flavonols, isoflavonoids, organic acids, and most of the phenolic acids showed increased accumulation in MT treatments as compared to CK. Whereas, coumarin, lignan, and free fatty acids accumulation were reduced in MT treatments as compared to CK (Additional file 1: Supplementary Table 3). This means during salt stress, the cotton seedlings produced higher coumarin as well as free fatty acids to cope with salt stress and upon the application of MT, the seedlings decrease coumarin biosynthesis, which showcases that seedlings are no more under stress [61, 62].

### The metabolite profile confirms transcriptome signatures related to phytohormone signaling, glutathione, and α-linoleic acid metabolism

We found that B1-treated seedlings had higher flavones, flavonols, vitamins, and organic acid contents. The reduced accumulation of indole-5-carboxylic acid and increased accumulation of isosalicylic acid O-glycoside and JA are consistent with the transcriptome findings that plant hormone signaling pathways is activated in response to MT treatment in salt stress cotton seedlings. The increased accumulation of glutathione in all the MT-treated cotton seedlings as compared to the CK affirms the transcriptome sequencing changes, indicating that glutathione accumulation is increased in response to MT treatment and the seedlings are relieved from salt stress (Additional file 1: Supplementary Table 3).

### Treatment-specificity metabolites

**Fig. 6:**
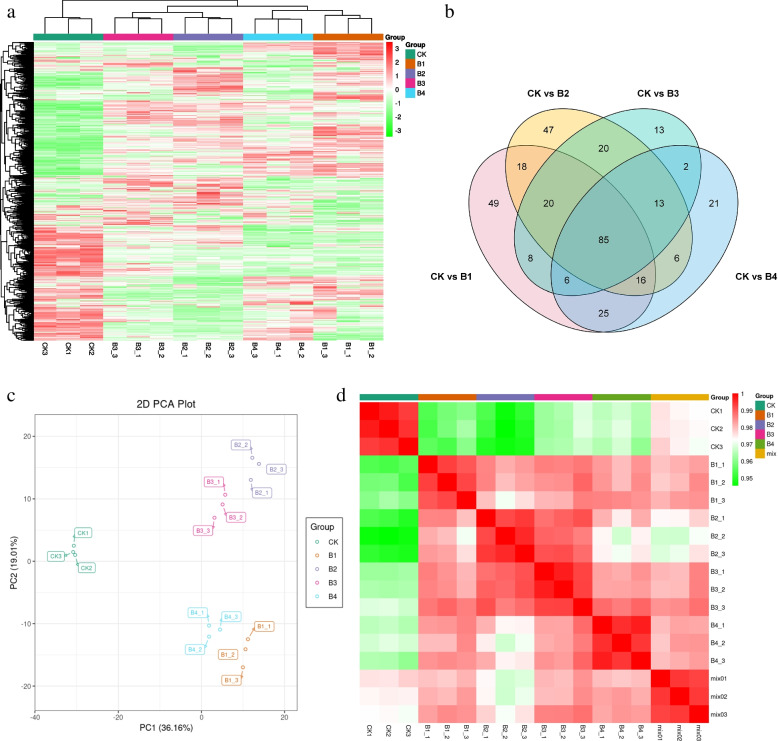
Top-10 accumulation metabolites in different treatment comparisons of the melatonin-treated cotton seedlings exposed to stress. The heatmap shows log2 foldchange values of the differentially accumulated metabolites, where red to green colors show increasing values. Where CK = 0.8% salt stressed, B1, B2, B3, and B4 = 0.8% salt-stressed seedlings exogenously sprayed with 100 ml of 50 μM, 100 μM, 200 μM, and 500 μM melatonin solution, respectively

Of the 49 CKvsB1 specific metabolites, 31 were accumulated in higher quantities in MT-treated seedlings as compared to CK. A major class of the upregulated DAMs was flavonoids (flavonols, flavanones, and flavones) followed by organic acids and phenolic acids. The top-10 accumulated metabolites in B1 as compared to CK are shown in Fig. 6. Twenty-one of the 47 CKvsB2 specific metabolites were accumulated in higher quantities in B2 as compared to CK; these were classified as amino acids and derivatives, saccharides and alcohols, alkaloids, organic acids, and flavonols. The most upregulated compound was N, N′-Diferuloylputrescine (Fig. 6). Thirteen DAMs were specific to CKvsB3; seven of which showed increased accumulation in B3 as compared to CK. The top-10 accumulated DAMs in B3 are presented in Fig. 6. Finally, 21 DAMs were specific to CKvsB4; nine of which were accumulated in higher quantities in B4 as compared to CK. Interestingly, the most up-accumulated metabolite in B3 and B4 was also N, N′-Diferuloylputrescine (Fig. 6). The most up-accumulation of N, N′-Diferuloylputrescine in B2, B3, and B4 as well as the 4th most up-accumulated metabolite in B1 shows that MT treatment increased the antioxidative activity in cotton seedlings as compared to CK. N, N′-Diferuloylputrescine is known for its antioxidative activities [63].

## Discussion

### Exogenous application of melatonin (100 mM) improves salt-stressed seedlings’ performance

Melatonin serves a range of biological functions in plants ranging from growth and development to enabling plants to withstand abiotic stresses [21]. In the present study, the upland cotton seedlings were exposed to salt stress and MT was applied exogenously. The morphological results indicated that MT-treated seedlings grew better than the control (stressed with salt but no MT application). These results are consistent with the earlier findings in this species that MT exerts positive effects on seed germination and growth [28, 35]. Our results that cotton seedlings performed better with the increase in MT concentration are important. Particularly, the result of the B2 (100 μM/L) seedlings performed significantly better than B1 and B3 and non-significantly different than B4 shows that this concentration is much more suitable for alleviating salt stress effects (Fig. 1). Similar results were observed in strawberries, where 100 μM/L MT imparted the best remedy effects on seedlings [64]. These findings deliver evidence that MT is a salt stress regulator in upland cotton at the seedling stage. Our findings also indicate that the higher concentrations of MT (200 μM/L and 500 μM/L) are not suitable and the salt stressed cotton seedlings are relatively less relieved from the stress as compared to B2. After germination, the seedling stage is the most important stage of the plant, and protecting it from stress ensures better yield [29], therefore, our results are important. Melatonin alleviates salt-stress effects in cotton seedlings by increasing expressions of photosynthesis, antenna proteins, and chlorophyll biosynthesis related genes.

Studies on different plant species have shown that these stress-alleviating effects of MT are due to the involvement of multiple signaling, growth, and homeostasis-related pathways [23, 25, 29, 32]. Therefore, in the current study, we employed a transcriptome sequencing and metabolome analysis approach to understand the key pathways that are involved in relieving upland cotton seedlings from salt stress. Firstly, the regulation of porphyrin and chlorophyll metabolism, chlorophyll biosynthesis, and photosynthesis of KEGG pathways-related genes is a clear indication of better plant performance in MT-treated seedlings as compared to CK. Most importantly, the expressions of hemF, hemH, hemL, and hemC indicate that the chlorophyll biosynthesis increased after MT treatment. In addition to chlorophyll biosynthesis, the Arabidopsis CLH1 was induced after tissue damage [65]. Thus, we can expect a similar function in cotton seedlings. The result that MT increased chlorophyll biosynthesis is further supported by the expressions of the antenna proteins (particularly of LHCB3 and 4, and LHCA2). The antenna proteins have been previously associated with the enhancement of salt stress tolerance by coordinating photosynthesis in Arabidopsis [66]. The upregulation of the photosynthesis-related proteins ATPF01, petJ, petF, petH, PsbP, PsbQ, and PsbR further confirms the role of MT in increasing the photosynthetic efficiency of the upland cotton seedlings during salt stress (Fig. 3) [67, 68]. Overall, we conclude that MT improves cotton seedling tolerance against salt stress by increasing the expressions of chlorophyll metabolism, antenna proteins, and photosynthesis pathway-related genes.

### Melatonin application triggers expression changes in plant-hormone signaling pathway

Melatonin’s growth-promoting activity is due to its auxin-like roles in plants such that its application slightly induces IAA. On the contrary, higher endogenous MT biosynthesis leads to lower IAA levels [69, 70]. Our results that AUX/IAA transcripts were downregulated in MT-treated seedlings (Additional file 1: Supplementary Table 2) are consistent with earlier reports in Arabidopsis that it can interfere in the auxin action [71]. Since ARFs e.g., ARF2 are reported to inhibit cell division [72], while ARF17 inhibits the expression of downstream proteins [73], therefore, it is possible that reduced expressions of ARFs in MT-treated seedlings indicate that the seedlings’ growth is improved as compared to CK. Other than auxin, cytokinins are known as senescence retardants. The upregulation of HKs in response to MT treatment shows that cytokinin is being perceived and participates in seedling growth as reported in Arabidopsis (Additional file 1: Supplementary Table 2) [74]. Abscisic acid biosynthesis decreases in salt-stressed *Cucumis sativus* with the application of MT [75], suggesting that its receptors’ expression should be decreased or no expression at all. Our observation that PYL4-like and PYL8-like showed reduced expressions as compared to CK in most of the MT treatments indicates that cotton seedlings produce a similar response under our experimental conditions. The reduced expression of the PP2Cs in all MT treatments further indicates that salt stress tolerance in upland cotton seedlings is enhanced upon MT application in an ABA-dependent manner [76]. Moving further in the plant-hormone signal transduction pathway, the downregulation of ERFs, ETRs, CTR1-lie, and EBF1 transcripts in MT treatments (Additional file 1: Supplementary Table 2) indicates an inhibitory effect on ethylene biosynthesis. Similar results were noted in lupin seedlings, where MT treatment caused inhibition of the rate of ethylene production [77]. Contrastingly, the upregulation of the brassinosteroid signaling-related genes is consistent with earlier reports that this hormone acts synergistically with MT and improves salt stress tolerance in cotton [23]. Additionally, the upregulation of CYCD3–3 transcripts in MT treatments helped cotton seedlings perform better as compared to CK (Additional file 1: Supplementary Table 2). This statement is based on the known function of CYCD3–3 in cell proliferation and secondary growth [57]. Finally, the differential expression of JA signaling-related transcripts indicates that MT treatment causes a stress-relieving effect on salt-stressed cotton seedlings [59]. These observations are also consistent with the differential accumulation of indole-5-carboxylic acid, isosalicylic acid O-glycoside, and JA in MT-treated seedlings as compared to CK. Overall, we conclude that the exogenous MT application significantly improves upland cotton seedlings’ tolerance to salt stress by activating a complex plant-hormone signal transduction cascade.

### Melatonin application increases flavonoid and linoleic acid contents in salt-stressed cotton seedlings

**Fig. 7:**
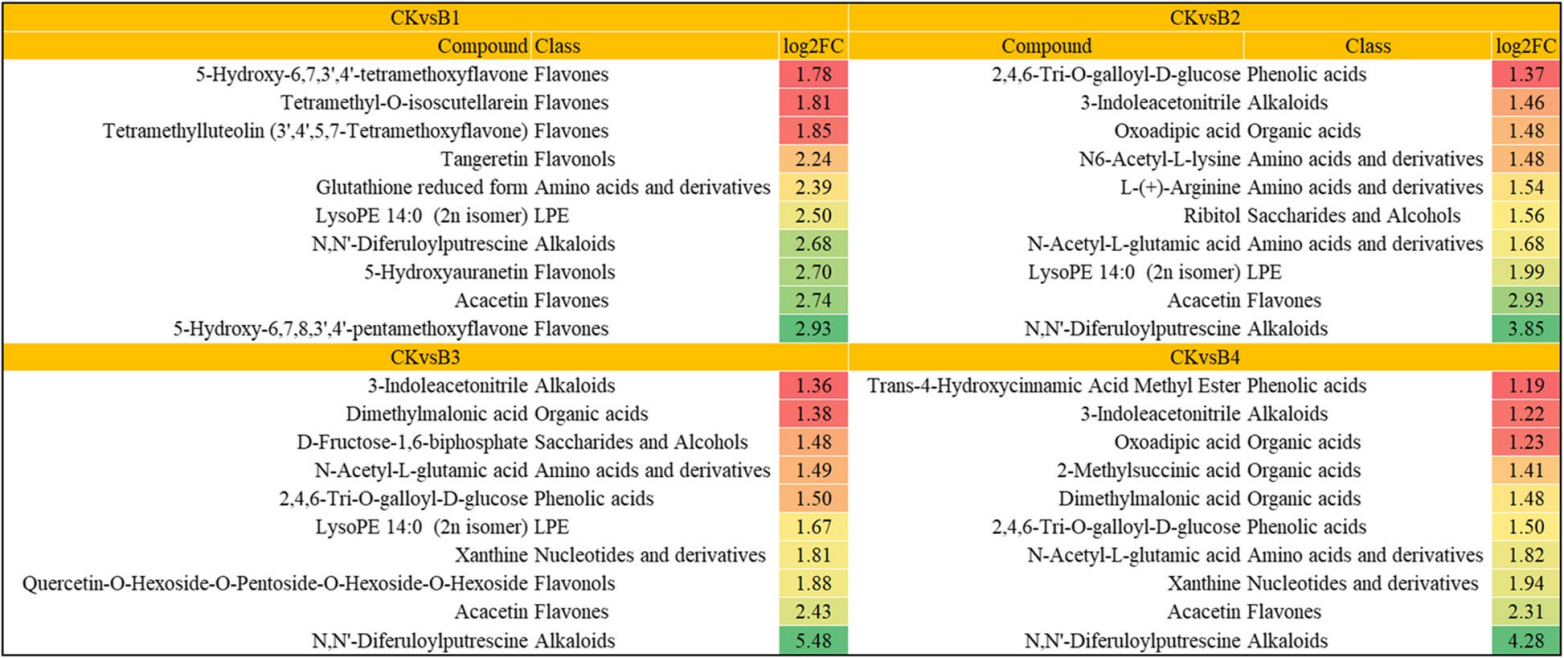
Heatmap of log2FC values of the DEGs enriched in linoleic acid metabolism (ko00591) and alpha-linoleic acid metabolism pathway (ko00592). The figure panel on the right shows the enriched DEGs (highlighted in red boxes/text) on the alpha-linolenic acid metabolism pathway (ko00592) The map was developed in the KEGG pathway database [54] using the KEGG pathway mapper [55] by searching Ko IDs of the DEGs on respective pathways. Where CK = 0.8% salt stressed, B1, B2, B3, and B4 = 0.8% salt-stressed seedlings exogenously sprayed with 100 ml of 50 μM, 100 μM, 200 μM, and 500 μM melatonin solution, respectively

When stressed seedlings are provided with the exogenous MT, the quantities of different metabolites such as flavonoids, linoleic acid, and other intermediates increase [78]. Our results that AOS, AOC, OPR, OPCL5, and ACX transcripts and increased accumulation of α-linoleic acid metabolism-related metabolites indicate their roles in relieving salt stress effects in upland cotton seedlings upon MT application (Fig. 7; Additional file 1: Supplementary Table 2–3). These observations are consistent with those reported in rice seedlings [31]. Particularly, the increased flavonoid biosynthesis indicates the reprogramming of the flavonoid biosynthesis pathway in response to MT treatment. This is evident from the higher accumulation of flavones, flavonols, and flavonoids (Fig. 6; Additional file 1: Supplementary Table 3) as well as the enrichment of large DEGs in related pathways.

### Melatonin application increases expressions of the antioxidative enzymes (genes) to alleviate salt-stress effects in cotton seedlings

Salt stress induces the production of ROS in cotton seedlings [79]. Upon MT treatment, the ROS scavenging cascades are activated and seedlings are protected from salt stress [80]. Our observations that GGT1, G6PD, ACA1, SOD, GST, and CAT-2 were upregulated in response to MT treatment as compared to CK indicate higher glutathione and L-glutamate biosynthesis (Fig. 4). This was confirmed in the metabolome analysis (Additional file 1: Supplementary Table 3). These observations are in line with the earlier reports that salinity induces ROS production, whereas MT treatment activates the antioxidant system [10]. This also means that MT increases the activities of the major protective antioxidant enzymes in cotton seedlings similar to many plant species e.g., soybean, maize, rice, cucumber, watermelon, and apple [21 and references therein]. The higher glutathione accumulation in MT-treated seedlings as compared to CK indicates lower oxidative denaturation of proteins and better salt stress tolerance [81]. Finally, the increased accumulation of N, N′-Diferuloylputrescine indicates improved antioxidative activities after MT treatment [63]. Taken together, we conclude that cotton seedlings, like many other plant species, recruit antioxidant pathway genes for ROS scavenging and protecting plants from salt stress.

### Melatonin applications increases expression of ion-homeostasis related genes in salt-stress cotton seedlings

Finally, the most important aspect of salt stress responses in plants is ion homeostasis. In this process, the major intracellular ions are Na^+^, K^+^, Ca^2+^, and H^+^. Among these, the Na^+^ enters plant cells during salt stress and denatures the cytosolic enzymes. To regulate this, plants need to maintain higher K^+^ and lower Na^+^ levels in the cytosol [82]. The specific expression of CHX2 in MT treatments and upregulation of other CHXs (CHX15 and CHX2) indicate Na^+^ exclusion under salt stress. Additionally, the better growth and performance by the MT-treated seedlings can also be due to the upregulation of PHO1 transcripts as enhanced salt tolerance in soybean was associated with higher PHO1 expressions [83]. Similarly, the increased expressions of AKT1 and SKD1 indicate that MT takes part in Na^+^/K^+^ homeostasis and enables upland cotton seedlings to withstand salt stress [84]. Our results also indicate that the SOS pathway is activated in MT-treated seedlings as evident from CIPK9-like and CHX7 gene expressions [85]. The downregulation of TPCs in MT treatments could indicate that the replenishing of the lost cytosolic K+ from vacuolar pools is achieved, while in CK it is still needed (Fig. 4) [86]. Overall, our results conclude that MT application enhances Na^+^/K^+^ homeostasis in cotton seedlings exposed to salt stress by changing the expressions of Na^+^ and K^+^ transporters [25, 87].

Considering that the MT application activated several key developmental, signaling, and ROS scavenging pathways, the future studies may also keep the most suitable MT content (100 μM and 500 μM) as a constant and test them against increasing NaCl concentrations. These experiments will also add up the knowledge on the key pathways involved in the MT application and upland cotton plant’s responses.

## Conclusion

**Fig. 8:**
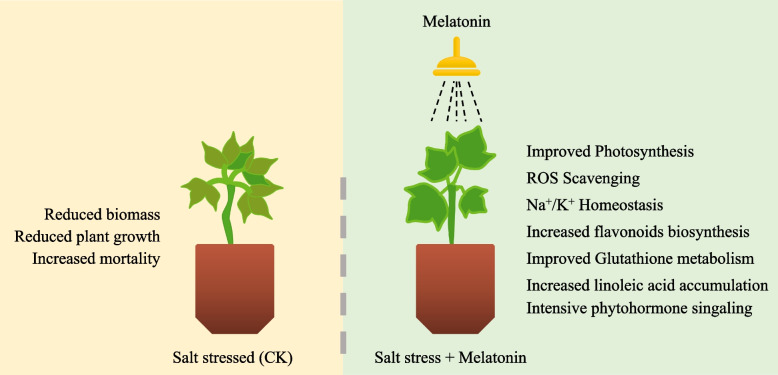
Schematic diagram showing melatonin application effects on salt stressed upland cotton seedlings. Exogenous melatonin application improved plant biomass and growth while reducing the mortality rate. The mechanisms that helped cotton seedlings to cope with salt stress include increased photosynthesis, ROS scavenging, Na^+^/K^+^ homeostasis, flavonoid, glutathione, and linoleic acid accumulation. Large-scale transcriptomic changes in the plant hormone signal transduction pathway were observed

Exogenous MT was sprayed on upland cotton seedlings exposed to salt stress and global metabolite and transcriptome changes were studied. Our results conclude that exogenous MT application increases seedling biomass and average plant growth and at the same time reduces mortality rate. The detailed transcriptome analyses conclude that chlorophyll metabolism and photosynthesis increase as evident by expression changes in related genes. Furthermore, linoleic acid biosynthesis is also increased in response to MT treatment. Extensive changes occur in the plant-hormone signaling pathway. Multiple ROS scavenging genes’ expressions increase leading to better plant growth and survival. This survival is further associated with improved homeostasis of Na^+^ and K^+^ in MT-treated seedlings as compared to CK. The increased accumulation of amino acids, flavones, flavonols, isoflavonoids, organic acids, and phenolic acids was measured in MT treatments as compared to CK. On the contrary, coumarin, lignan, and free fatty acids accumulation were reduced in MT treatments as compared to CK. Overall, we conclude that MT is a salt stress regulator in upland cotton at the seedling stage (Fig. 8).

## Supplementary Information

**Additional file 1 Supplementary Table 1.** Summary of cotton seedlings RNA sequencing challenged with salt stress and treated with different melatonin concentrations. **Supplementary Table 2.** Differentially expressed genes involved in salt stress tolerance in upland cotton seedlings sprayed with melatonin. **Supplementary Table 3.** Differentially accumulated metabolites in cotton seedlings challenged with salt stress and exogenously treated with melatonin.

**Additional file 2 Supplementary Fig. 1.** A. qRT-PCR analysis of the selected genes in upland cotton challenged with drought stress. Where, B1, B2, B3, and B4 = 0.8% salt-stressed seedlings exogenously sprayed with 100 ml of 50 μM, 100 μM, 200 μM, and 500 μM melatonin solution, respectively. The x-axis and y-axis represent treatments and relative gene expressions, respectively. The bars are mean relative expression values of three replicates. The error bars represent standard deviation. B. The Pearson correlation between the gene expression changes based on qRT-PCR and RNA-seq.

**Additional file 3 Supplementary Fig. 2**. KEGG scatter plots show pathways to which the differentially accumulated metabolites were enriched. (PPTX 821 kb)

## Data Availability

The raw transcriptome data has been submitted to NCBI SRA under the accession number: PRJNA856850 (https://www.ncbi.nlm.nih.gov/bioproject/PRJNA856850).

## Abbreviations

MT: Melatonin
ROS: Reactive oxygen species
OPR: 12-oxophytodienoate reductase 2-like
cGMP: 3,5-cyclic guanosine monophosphate
MEP2: 3-hydroxy acyl-CoA dehydrogenase
OPLC5: 4-coumarate-CoA ligase-like 5
ABA: abscisic acid
ABF7: ABSCISIC ACID-INSENSITIVE 5-like protein 7
AOC: allene oxide cyclase
AOS: allene oxide synthase-like
ACA1: alpha carbonic anhydrase 1
APX: ascorbate peroxidase
AUX1: auxin influx carrier
SAUR: auxin-responsive proteins SUAR
BRI1: brassinosteroid insensitive 1
BKI1: BRI1 kinase inhibitor 1
CBL: calcineurin B-like proteins
CAT: catalase isozyme 2-like
CCX: cation/calcium exchanger 2-like
CHX: cation/H+ antiporters
CIPKs: CBL-interacting protein kinases
LHCB4: chlorophyll a-b binding protein CP29.3
hemF: coproporphyrinogen-III oxidase 1
CYCD3–1: cyclin-D3–1-like
CYCD3–3: cyclin-D3–3-like
petJ: Cytochrome c6
HKs: cytokinin receptor histidine kinases
DAMs: Differentially accumulated metabolites
DEGs: Differentially expressed genes
DETs: Differentially expressed transcripts
EBF1: EIN3-binding F-box protein 1-like
ETR: ethylene receptors
ERFs: ethylene response factors
petF: Ferredoxin
petH: ferredoxin--NADP+ reductases
hemH: ferrochelatase-2
FPKM: Fragments Per Kilobase of transcript per Million fragments mapped
ATPF0A: F-type H + -transporting ATPase
GO: Gene Ontology
G6PD: glucose-6-phosphate 1-dehydrogenase
hemL: glutamate-1-semialdehyde 2,1-aminomutase 2
GGT1: glutamyltranspeptidase 1-like
GPX: glutathione peroxidase
GST: glutathione s-transferase
GH3: IAA-amido synthetase
IAA: indole-3-acetic acid
JAOMT: Jasmonate O-methyltransferase
JA: Jasmonic acid
KEA: K+ efflux antiporter
KEGG: Kyoto encyclopedia of genes and genomes
PEPA: leucine aminopeptidase 3
ChlI: magnesium-chelatase
NHX7: Na+/H+ exchanger 7
CK: Negative control
NO: nitric oxide
OPLS-DA: Orthogonal partial least squares discriminant analysis
PCC: Pearson’s Correlation Coefficient
ACX: peroxisomal acyl-coenzyme A oxidase 1-like
PLA2: phospholipase A2
LHCA2: photosystem I chlorophyll a/b-binding protein 6
PsbR: photosystem II 10 kDa protein
PsbS: photosystem II 22 kDa proteins
PsbP: photosystem II oxygen-evolving enhancer protein 2
PsbQ: photosystem II oxygen-evolving enhancer protein 3
hemC: porphobilinogen deaminase
CK+: Positive control
PCA: Principal Component Analysis
PP2C: protein phosphatase 2C
qRT-PCR: Quantitative Real time PCR
RBOHs: RESPIRATORY BURST OXIDASE HOMOLOGs
SOS: Salt overlay sensitive
CTR1-like: serine/threonine-protein kinase CTR1-like
SnRK-like: serine/threonine-protein kinase SAPK2-like
SOD: superoxide dismutase
SKD1: SUPPRESSOR OF K+ TRANSPORT GROWTH DEFECT 1
TF: Transcription factors
ARR/ORR: two-component response regulators
CAX3: vacuolar cation/proton exchanger 3
VIP: Variable importance of projection
