# Supplementary figures and images for: Combined transcriptome and metabolome analysis revealed pathways involved in improved salt tolerance of Gossypium hirsutum L. seedlings in response to exogenous melatonin application
Source: BMC Plant Biol. 2022 Nov 30;22:552. doi: 10.1186/s12870-022-03930-0 (PMC9710056; doi:10.1186/s12870-022-03930-0)

Figure S1A

Relative gene expression

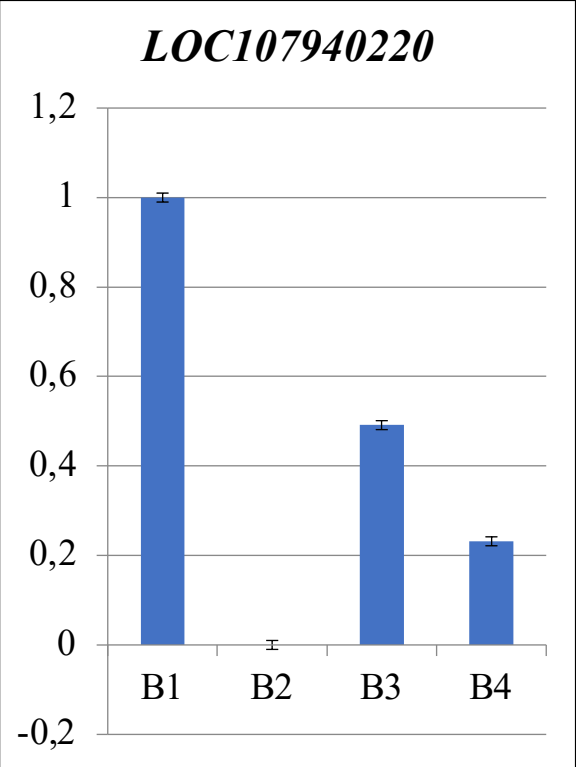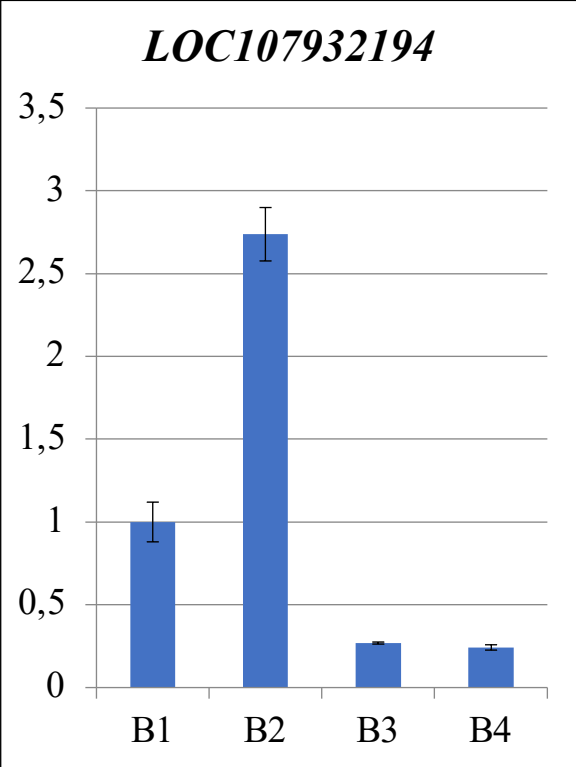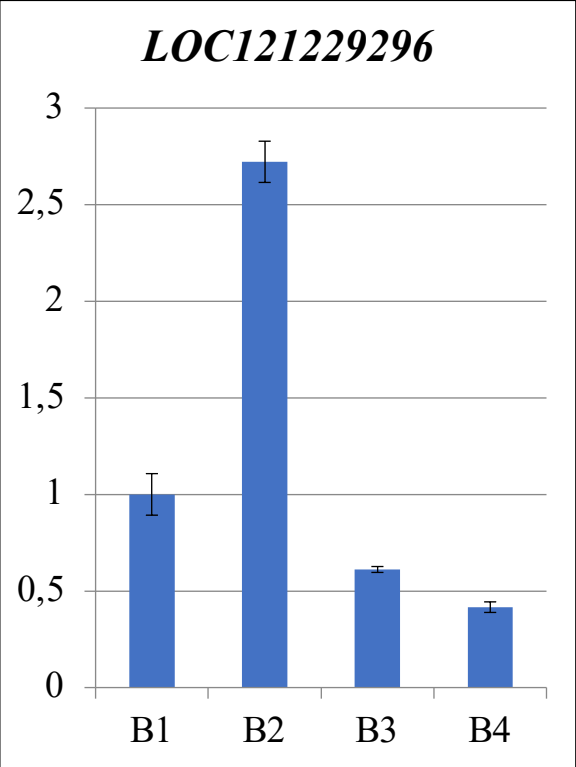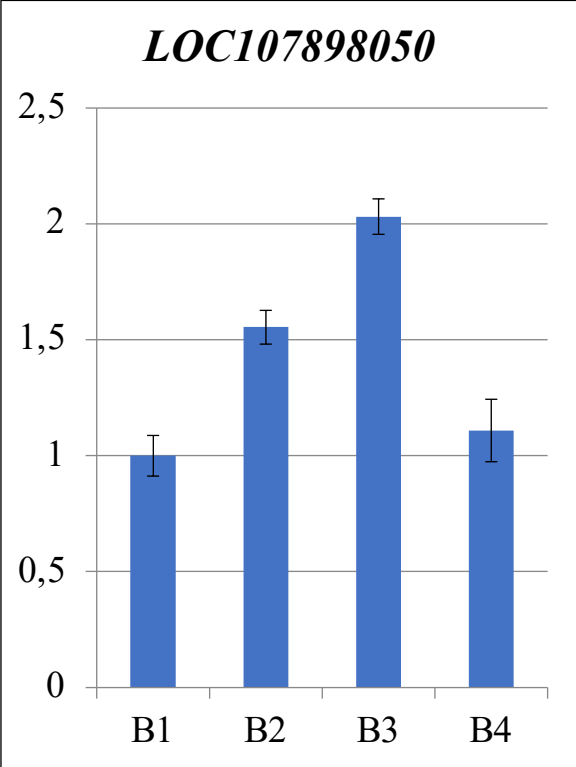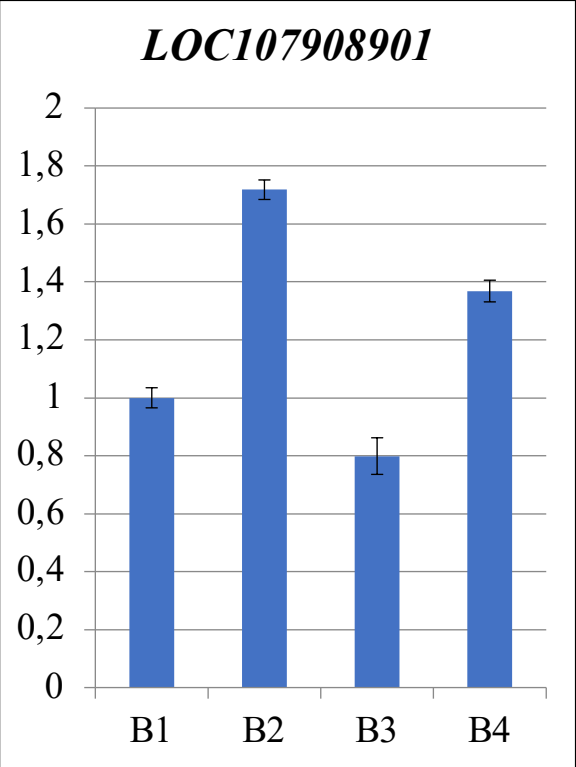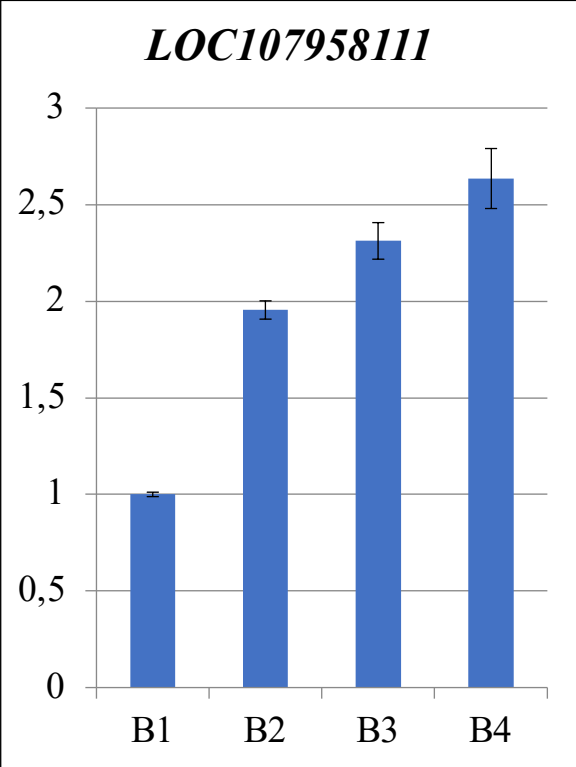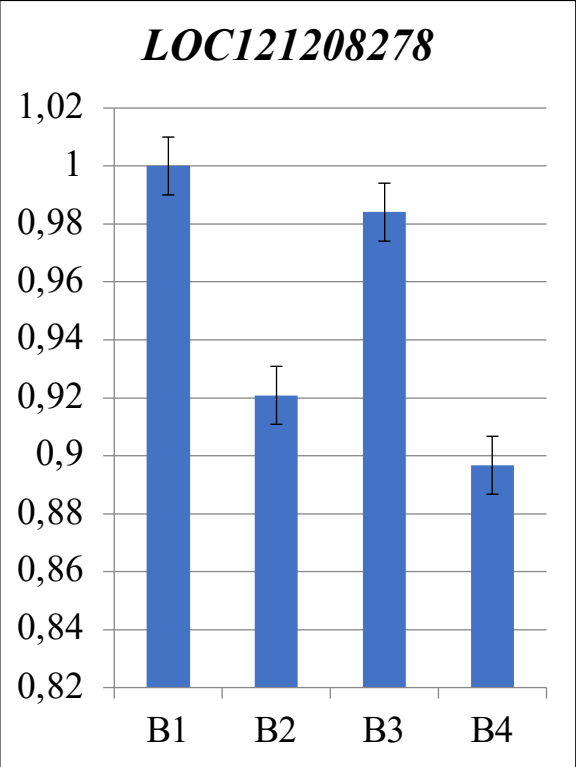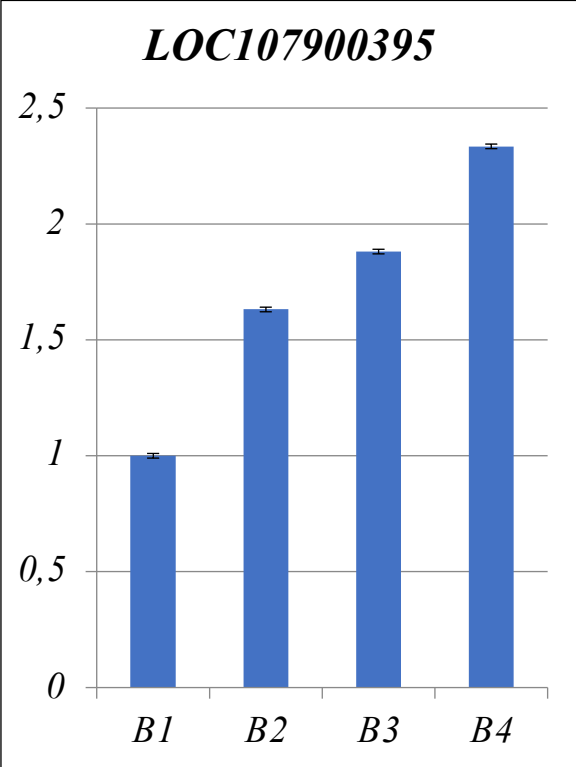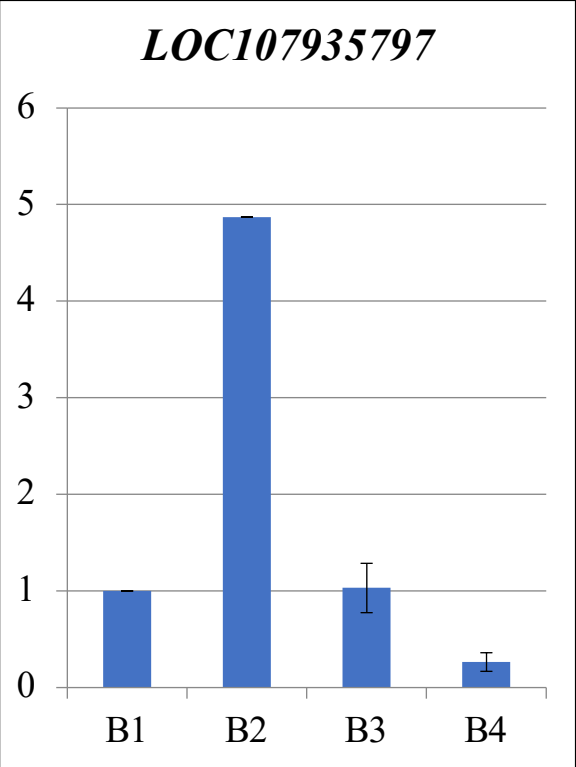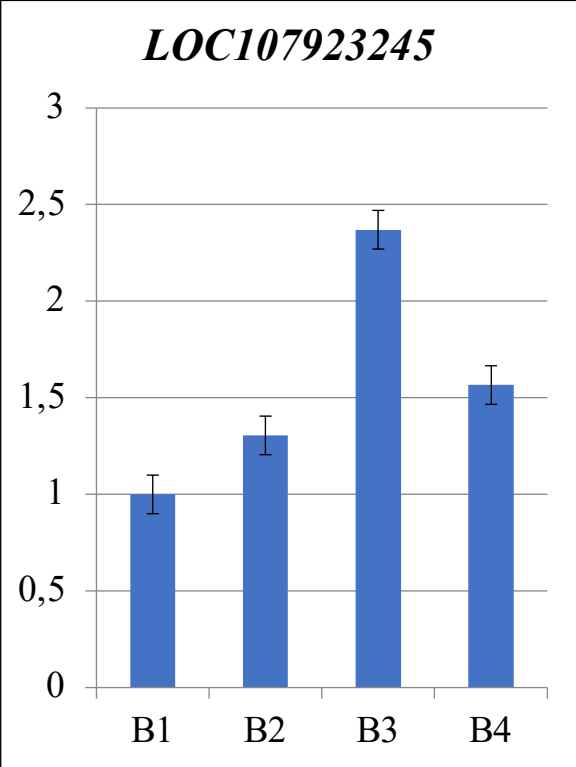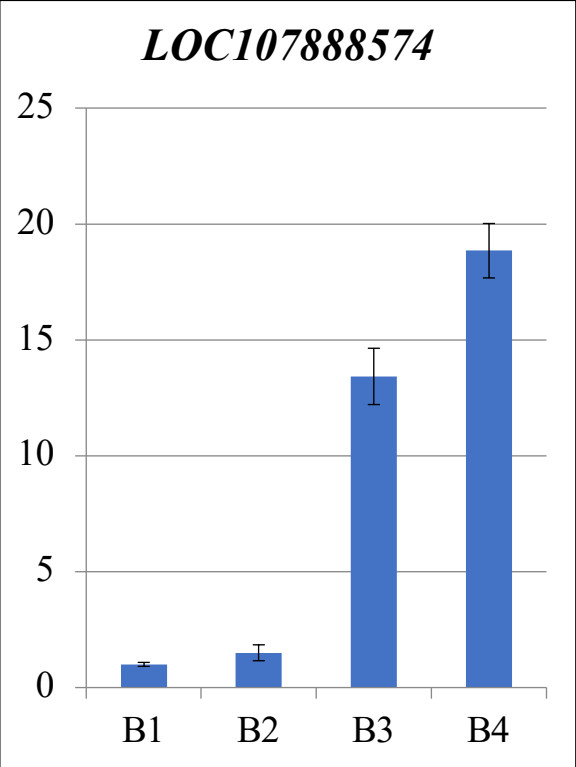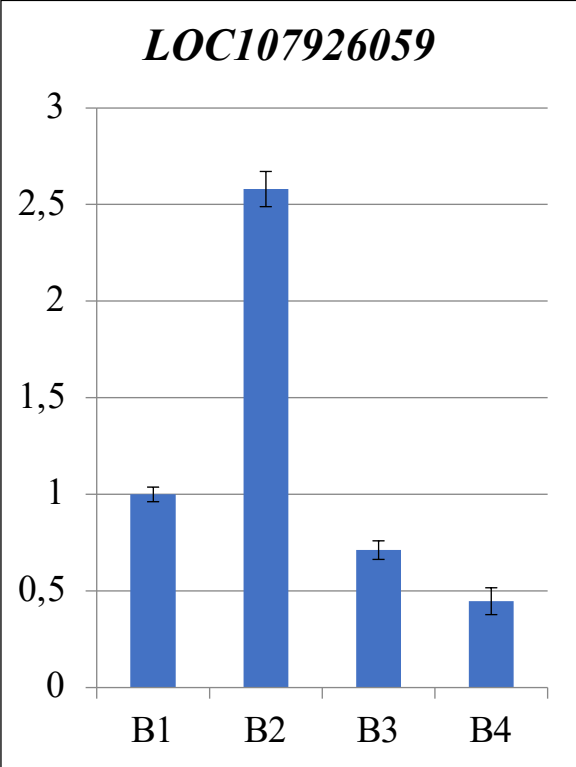

Figure S1B

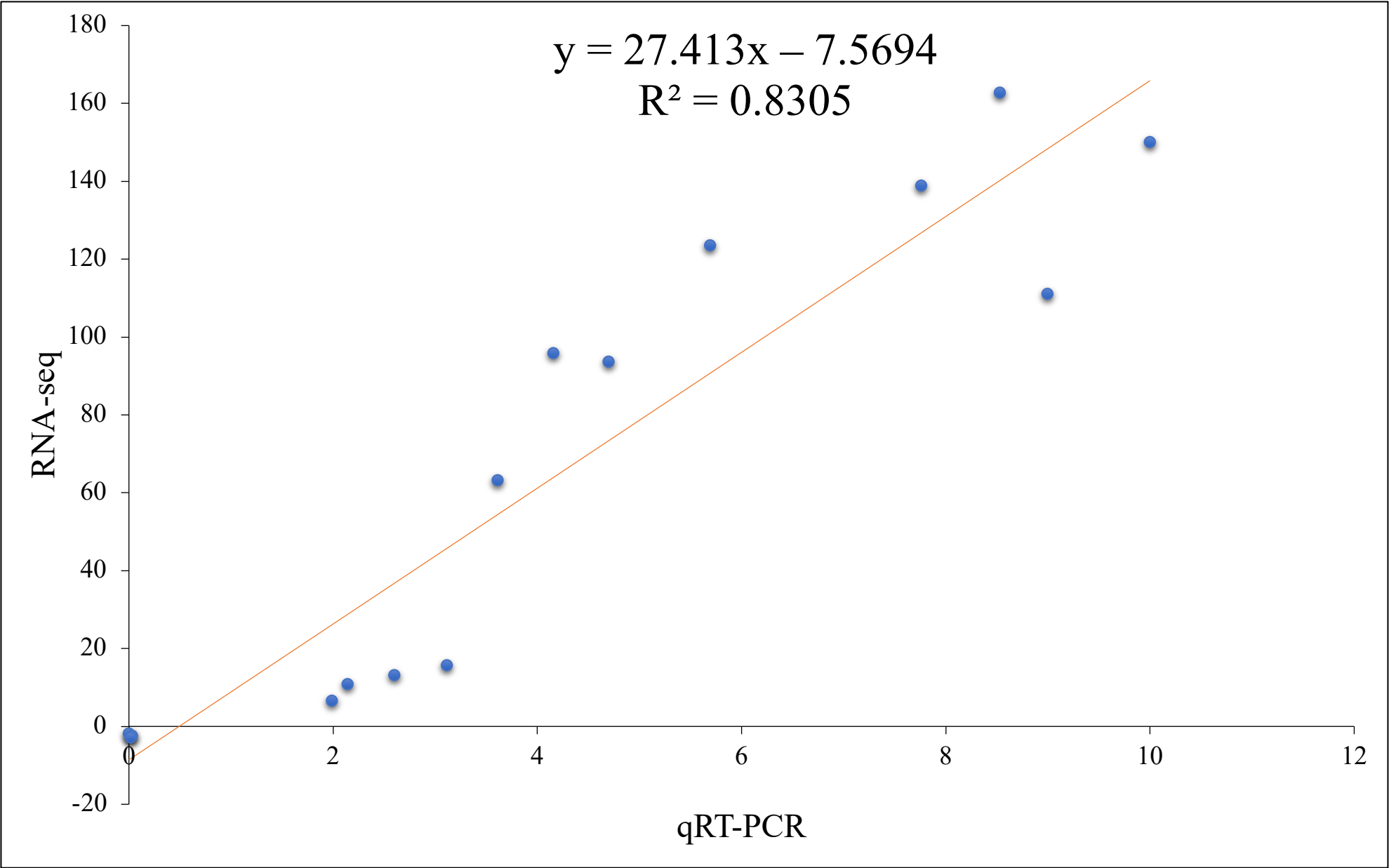

Supplement: Supplementary file 2 — Additional file 2 Supplementary Fig. 1. A. qRT-PCR analysis of the selected genes in upland cotton challenged with drought stress. Where, B1, B2, B3, and B4 = 0.8% salt-stressed seedlings exogenously sprayed with 100 ml of 50 μM, 100 μM, 200 μM, and 500 μM melatonin solution, respectively. The x-axis and y-axis represent treatments and relative gene expressions, respectively. The bars are mean relative expression values of three replicates. The error bars represent standard deviation. B. The Pearson correlation between the gene expression changes based on qRT-PCR and RNA-seq. [file 12870_2022_3930_MOESM2_ESM.pdf]

## Slide 1
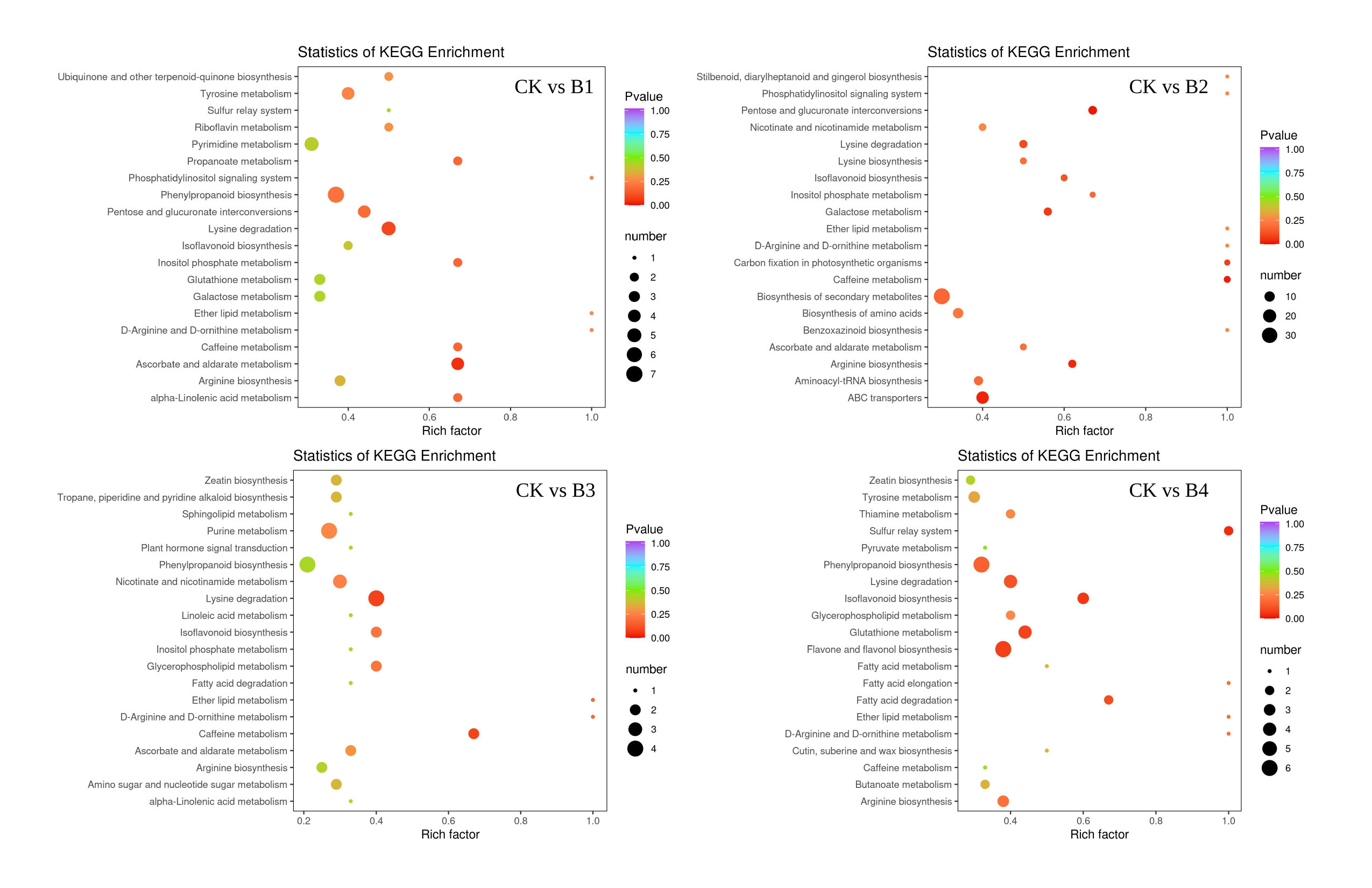

CK vs B1
CK vs B2
CK vs B3
CK vs B4

Supplement: Supplementary file 3 — Additional file 3 Supplementary Fig. 2. KEGG scatter plots show pathways to which the differentially accumulated metabolites were enriched. (PPTX 821 kb) [file 12870_2022_3930_MOESM3_ESM.pptx]
